# Find the Flame: Predictive Biomarkers for Immunotherapy in Melanoma

**DOI:** 10.3390/cancers13081819

**Published:** 2021-04-10

**Authors:** Mattia Garutti, Serena Bonin, Silvia Buriolla, Elisa Bertoli, Maria Antonietta Pizzichetta, Iris Zalaudek, Fabio Puglisi

**Affiliations:** 1 CRO Aviano National Cancer Institute IRCCS, 33081 Aviano, Italy; elisa.bertoli@cro.it (E.B.); pizzichetta@cro.it (M.A.P.); fabio.puglisi@uniud.it (F.P.); 2 DSM—Department of Medical Sciences, University of Trieste, 34123 Trieste, Italy; sbonin@units.it; 3 Department of Medicine (DAME), University of Udine, 33100 Udine, Italy; silvia.buriolla@cro.it; 4 Dipartimento di Oncologia, Azienda Sanitaria Universitaria Friuli Centrale, 33100 Udine, Italy; 5 Department of Dermatology, University of Trieste, 34123 Trieste, Italy; Iris.zalaudek@gmail.com

**Keywords:** biomarkers, immunotherapy, checkpoint inhibitors, PD-1, PDL-1, CTLA-4, nivolumab, pembrolizumab, meta-analysis, melanoma

## Abstract

**Simple Summary:**

Immunotherapy has revolutionized the therapeutic landscape of melanoma. However, the absence of clinically validated predictive biomarkers is one of the major causes of unpredictable efficacy of immunotherapy. Indeed, the availability of predictive biomarkers could allow a better stratification of patients, suggesting which type of drugs should be used in a certain clinical context and guiding clinicians in escalating or de-escalating therapy. However, the difficulty in obtaining clinically useful predictive biomarkers reflects the deep complexity of tumor biology. Herein, we review the available literature to depict the most useful or promising biological biomarker able to predict immunotherapy response in melanoma. We also make a meta-analysis regarding PDL1 expression in melanoma and immune checkpoint response.

**Abstract:**

Immunotherapy has revolutionized the therapeutic landscape of melanoma. In particular, checkpoint inhibition has shown to increase long-term outcome, and, in some cases, it can be virtually curative. However, the absence of clinically validated predictive biomarkers is one of the major causes of unpredictable efficacy of immunotherapy. Indeed, the availability of predictive biomarkers could allow a better stratification of patients, suggesting which type of drugs should be used in a certain clinical context and guiding clinicians in escalating or de-escalating therapy. However, the difficulty in obtaining clinically useful predictive biomarkers reflects the deep complexity of tumor biology. Biomarkers can be classified as tumor-intrinsic biomarkers, microenvironment biomarkers, and systemic biomarkers. Herein we review the available literature to classify and describe predictive biomarkers for checkpoint inhibition in melanoma with the aim of helping clinicians in the decision-making process. We also performed a meta-analysis on the predictive value of PDL-1.

## 1. Introduction

Historically, melanoma was described as a highly lethal disease [1]. However, in the last 10 years, several new therapeutic strategies have been shown to increase survival in the metastatic and adjuvant settings [2,3,4]. Among the contemporary available treatments, immunotherapy (e.g., checkpoint inhibitors (ICIs)) has demonstrated an impressive efficacy. Indeed, checkpoint inhibition, which was described as “breakthrough of the year 2013” [5], has also shown to be able to control melanoma in a long-term fashion [4]. These long-term remissions have opened up the possibility of a virtual curative effect of immunotherapy. However, to date, reliable biomarkers able to predict checkpoint inhibition efficacy in melanoma are lacking.

The availability of predictive biomarkers for immunotherapy could help clinicians choose the best therapeutic strategy for melanoma patients. Practically, where a benefit from immunotherapy is predicted, treatment de-escalation might be possible. On the contrary, melanomas predicted to be refractory to checkpoint inhibition could benefit from alternative treatments (i.e., targeted therapies) or a treatment escalation. Moreover, in some patients treated with immunotherapy, a precocious progression of the disease has been described. Those patients who are called hyper-progressors seem to have a poor prognosis [6]. Predictive biomarkers could help to identify these patients and treat them accordingly.

Predictive biomarkers for immunotherapy can be classified into three major groups: tumor-intrinsic biomarkers, which are expressed by tumor cells (e.g., PD-1/PDL-1, tumor mutational burden); tumor microenvironment biomarkers (e.g., tumor-infiltrating lymphocyte); and systemic biomarkers (e.g., circulating factors, microbiota).

Herein we review the available literature to classify and describe predictive biomarkers for checkpoint inhibition in melanoma to help clinicians in the decision-making process (Table 1). In particular, we focus on neoadjuvant and metastatic settings, where an evaluation of response rate can be performed. Moreover, we consider trials with anti-PD-1/PDL-1 and/or anti-CTLA-4.

## 2. Materials and Methods

### 2.1. Searching Details

For the meta-analysis we searched the database PubMed using the following keywords: (1) PD-L1 expression melanoma immunotherapy response; (2) melanoma keynote; (3) PD-L1 immunohistochemistry melanoma predictive; (4) PD-L1 nivolumab response melanoma; (5) PD-L1 expression predictive immunotherapy melanoma. We searched articles published up to November 2020. The reference lists were also carefully checked to identify additional eligible studies. All analyses were carried out with previously published data; consequently, no ethical approval or patient consent was necessary in this study. For the other manuscript sections, we performed scoping research.

### 2.2. Selection Criteria

Studies were included if they met the following criteria: (1) inclusion of patients diagnosed with confirmed melanoma; (2) treatment with immune checkpoint inhibitors; (3) detection of PD-L1 expression in the melanoma tissue, including metastases, by immunohistochemistry (IHC) irrespective of the antibody clone used; (4) identification of a definite cutoff value in the analysis of PD-L1 expression; (5) availability of RECIST 1.1 data (responders = complete + partial response according to RECIST for solid tumors) in PD-L1-positive and in PD-L1-negative patients; (5) publications in English language. Determination of PD-L1 expression in melanoma was based on most analyzed studies by the percentage of PD-L1 expressing tumor cells; however, in a minority of studies, inflammatory cells in nest of tumor cells were also counted. 

The exclusion criteria were as follows: (1) case reports, reviews (systematic reviews were retained), letters, and correspondences; (2) studies without available or usable information; (3) studies including adjuvant therapy with immune checkpoint inhibitors; (4) studies lacking PD-L1 or RECIST data; (5) animal studies; (6) publications in languages other than English; (7) duplicate studies.

### 2.3. Data Extraction

One researcher (S.B.) extracted basic information from the included studies, also analyzing supplementary data where available. The following data were extracted from eligible studies: the first author’s name, publication year, sample size, detection method, antibody used (where available), treatment, cutoff values, and number of responders and non-responders in the group of PD-L1-positive and PD-L1-negative patients.

### 2.4. Statistical Analysis

The percent of responders and non-responders in the group of PD-L1-positive and PD-L1-negative was used to set up the meta-analysis for binary data. The association between PD-L1 expression and response to ICI therapy was assessed by odds ratios (ORs) and their 95% CIs. The *I*^2^ metric was used to inspect the statistical heterogeneity of the data. A *p* value of less than 0.1 or an *I*^2^ value of more than 50% indicated significant heterogeneity, and a random effects model was employed for calculation. All statistical analyses were carried out using Stata version 16.0 (STATA Corp., College Station, TX, USA). *p* < 0.05 was considered to indicate statistical significance.

### 2.5. Results

In PubMed, after removal of duplicates, 309 publications were retained. From these, 52 articles were read as full text. Of these, 22 articles were retained as they fulfilled the inclusion criteria.

## 3. Tumor-Intrinsic Biomarkers

### 3.1. Tumor Mutational Burden

Tumor mutational burden (TMB) is an index that summarizes the mutational load of a tumor [84]. Since a high number of mutations could translate into a high number of neoantigens that the immune system can recognize, it has been hypothesized that TMB could act as a proxy for ICI effectiveness.

The definition and the methods for the detection of TMB are evolving [85]. Initially, the tumor mutational load was searched in the tumor specimen through the costly whole-exome sequencing (WES) technique, which takes into account the non-synonymous mutations in the coding regions of the genome [86]. More recently, to reduce costs and increase the breadth of investigation, next-generation sequencing (NGS) has been used on restricted gene panels in place of WES [10,14,87]. Among NGS methods, two tests have recently received approval from the FDA: FoundationOne CDx [88] (Cambridge, USA) and MSK-IMPACT [89] (Memorial Sloan Kettering, USA). The first one profiles 324 genes (corresponding to 1.1 Mb of coding genome) and also includes short indel in intronic regions, which are excluded from whole-exome sequencing. MSK-IMPACT analyzes 468 cancer-related genes for exonic mutations (approximately 1.2 Mb). More recently, in an attempt to simplify the search for TMB, a liquid biopsy approach has been used. Although at the beginning a discordance between tissue- and blood-based approaches seemed evident [90], a convergence of results might have been reached [91,92,93]. The detection of TMB in liquid biopsy could offer several advantages over the tissue-based approach. For example, it is a non-invasive procedure that allows serial sampling over time and the detection of TMB status in patients who do not have adequate tissue available.

Independently from the method used to analyze TMB, a linear correlation between TMB levels and the efficacy of ICIs seems to exist [94]. However, there is still a lack of consensus regarding the cutoff for the definition of high (TMB-H), intermediate(TMB-I), and low levels (TMB-L) of TMB. For example, FoundationOne CDx uses the threshold of 20 mutations/Mb for the definition of TMB-H, 6–19 mutations/Mb for the TMB-I, and ≤5 mutations/Mb for TMB-L [88,95]. Nonetheless, the FDA approved the agnostic use of pembrolizumab for tumors with a TMB threshold of at least 10 mutations/Mb. Moreover, since the mutational load varies considerably between histologies, the use of the top 20% value of TMB for each histology has been proposed [89]. However, a harmonization between methodologies is highly needed and is ongoing [94,96].

The observation that TMB levels show a linear correlation with the response to ICIs seems quite logical since a higher number of mutations could translate into a higher number of neoantigens that the immune system could recognize. However, this syllogism is somehow imperfect, as even tumors with a TMB-H have a response rate (RR) to ICIs of only 45% [97]. A possible explanation might reside in the variable presentation of neoantigens to lymphocytes that is strictly correlated to the proper functioning of MHC machinery (see below).

In melanoma, coherently with other cancer types, TMB levels correlate to ICI RR both with anti-PD-1/PDL-1 and anti-CTLA-4 therapies. As shown by Cristescu et al. [13], melanomas with TMB-H have a RR to pembrolizumab of 42% versus only 9% of melanomas with TMB-L. However, in that study, a T-cell inflamed gene-expression profile emerged as another variable influencing RR to pembrolizumab, suggesting that TMB might be used in conjunction with other biomarkers to predict ICI response better. Similar results have been shown by Van Allen et al. for ipilimumab [7].

Even though TMB-H alone can identify a subset of tumors particularly sensitive to ICIs, it should be highlighted that TMB-L cancers could also derive benefits from immunotherapies, albeit in a small percentage of patients (5%) [97]. Theoretically, even in a context of low mutational burden, strong immunogenic neoantigens could be generated, albeit with a lower probability compared to a TMB-H context.

### 3.2. MHC-I and MHC-II

Major histocompatibility complex (MHC) is the cellular machinery that is exposed on the cellular surface of small peptides that can be recognized by immune cells [98,99]. These, in turn, could become activated if the peptides that they recognize are identified as “non-self”. MHC can be classified as type I and type II [98,99]. The first can mediate the activation of CD8 lymphocytes subpopulation, while MHC class II regulates the activation of CD4 lymphocytes.

As stated above, the roles of TMB and MHC are quite intertwined. In order to be recognized by the immune system, a neoantigen has to be exposed on a cancer cell surface through the MHC machinery. While a high TMB increases the chance to generate immunogenic neoantigens, a functioning MHC machinery increases the chance that these immunogenic neoantigens will be identified by immune cells. The type of immune response after the recognition of the neoantigen by the immune cells may vary. CD8 lymphocytes, for instance, can exert a direct cytotoxic activity against cancer cells, while CD4 lymphocytes can stimulate a broad inflammatory response through the production of interferon-gamma [98,99].

In melanoma, several pieces of evidence show a predictive role for MHC to immunotherapy. However, a different predictive role between MHC class I and II seems to exist. In the work of Johnson et al., MHC class II positive-melanomas have a better response rate, progression-free survival, and overall survival in anti-PD-1 therapy [18,19]. However, as shown by Liu et al., the predictive role of MHC class II expression in melanoma cells in anti-PD-1 might be restricted in patients with previous exposure to an anti-CTLA-4 [17]. Interestingly, Rodig et al. showed a differential predictive role in immunotherapy for MHC class I and II [20]. While class II seems to predict benefit from anti-PD-1 therapy, MHC class I could be predictive for anti-CTLA-4 drugs. Although checkpoint inhibitors’ anticancer effect has historically been linked to the activation of cytotoxic CD8 lymphocytes [100], mounting evidence highlights a pivotal role for CD4, especially during an anti-PD-1 therapy [101]. Finally, the loss of B2M, a crucial protein belonging to the MHC class I system, has been described as a resistance mechanism to both anti-CTLA-4 and anti-PD-1 drugs [21].

Of note, there is still a lack of consensus regarding the cutoff for the definition of high- or low-MHC expressing tumors. Moreover, several proteins belonging to MHC machinery could be evaluated, and this could jeopardize the identification of MHC-positive or -negative cancers [98,99].

### 3.3. PD-1/PDL-1

**Figure 1:**
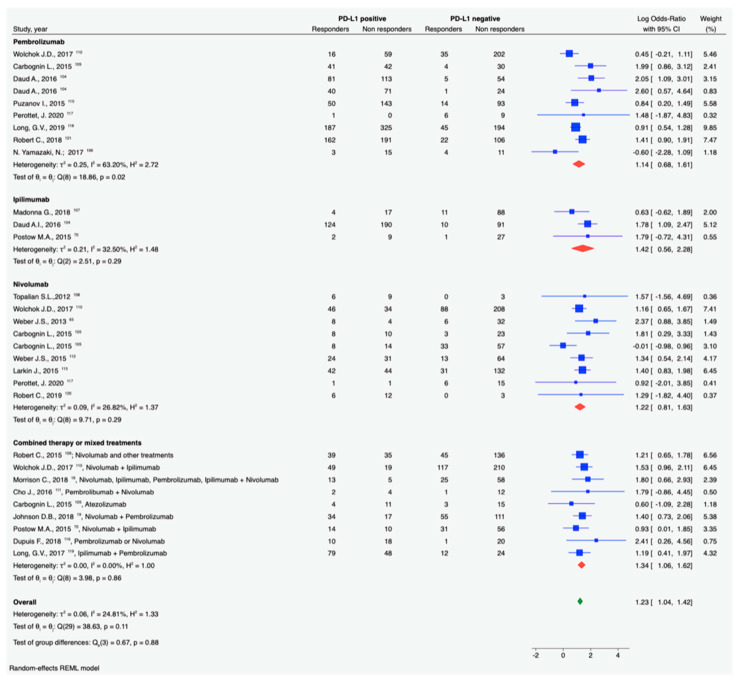
Forest plots of odds ratio (in log scale) of response to ICI (checkpoint inhibitor) in metastatic or non-resectable melanoma patients according to positivity to PD-L1 by immunohistochemistry. Meta-analysis was sub-grouped according to ICI treatment. The size of squares mirrors the weight of the study in the meta-analysis.

Programmed cell death 1 receptor (PD-1) is a checkpoint molecule present on T-cells, B-cells, and natural killer cells (NKs), which can interact with its ligands: PD-L1, expressed on tumor cells, and PD-L2, which is mainly present on hematopoietic cells [102]. The binding of PD-1 with its ligands is responsible for T-cell dysfunction and/or neutralization. Thus, overexpression or amplification of PD-L1 by tumor cells possibly diminishes immune antitumor response [102] with a mechanism of adaptive resistance [103]. Therefore, theoretically, patients whose tumors have a high level of PD-L1 should benefit better from ICI therapy, especially for those drugs targeting the PD-1/PD-L1 axis. PD-L1 is, indeed, the most commonly recognized biomarker to predict immunotherapy response in patients with different solid tumors, including cutaneous melanoma [104]. Immunohistochemical tests to assess PD-L1 expression are approved by the US Food and Drug Administration (FDA) as companion diagnostic assays for immunotherapy response for several solid tumors, but not for melanoma [105,106]. Staining issues related to specific antibody clones (28-8 vs. 22C3) as well as staining protocols have been successfully fixed, and PD-L1 staining has been highly harmonized and validated [107]. Nonetheless, immunohistochemistry using clone SP142 returned the lowest positivity level in most solid tumors [108]. In melanoma, positivity cutoffs for PD-L1 are 1% (corresponding to Melscore 2) [109] or 5% of tumor cells [110]. Alternatively, in addition to tumor cells, inflammatory cells in nests of tumor cells have been sometimes included in the count [111]. Rarely, melanin pigment in primary or metastatic melanomas can impact PD-L1 immunohistochemistry interpretation [108]. The meta-analysis carried out in the present review was set using as the endpoint of RECIST response rate (complete or partial response) to ICI therapy with respect to the PD-L1 expression by immunohistochemistry irrespective of the antibody clone and the cutoff (details of the inclusion criteria are reported in the Supplementary Material). Using selection criteria, 23 studies, including results for 4710 patients, were retained for the analysis [16,19,43,44,45,46,47,70,75,109,110,111,112,113,114,115,116,117,118,119,120,121]. Despite the heterogeneity in the treatments and staining cutoffs, our analysis shows that patients with PD-L1-positive melanomas/recurrences have, on average, a higher rate of response to ICI, as shown in Figure 1 and Table 2. By sorting data per ICI treatment, no significant difference in the overall response rate between PD-L1-positive and -negative melanomas was observed for ipilimumab treated patients, in line with the fact that it is an anti-CTLA-4 molecule. However, for pembrolizumab, nivolumab, and combined regimens, there is a significantly higher benefit in patients with positive PD-L1 melanomas. Notwithstanding, PD-1/PD-L1 inhibitors appear to also have activity in subsets of patients who do not meet IHC positivity to PD-L1 [122]. This is mainly because the PD-L1/PD-1 axis is not the only player in response to checkpoint inhibition [103]. It is manifest that PD-L1 status alone cannot be used as a reliable biomarker of therapeutic response to anti-PD-1 immunotherapy in melanoma. PD-L1 expression is dynamic and transient with possible intra-patient and intratumor heterogeneity [123], and it can be affected by many factors, including previous therapies and the presence of tumor-infiltrating immune cells [109]. Therefore, in melanoma, PD-L1 status should be combined with other criteria, such as TMB [104], CD8, and PD-1 in T-cells [124], to better describe the response to PD-1/PD-L1 inhibitors.

## 4. Microenvironment Biomarkers

### 4.1. TILs

Tumor-infiltrating lymphocytes (TILs) are a heterogeneous population comprising several cell subtypes such as effector (CD8+) and regulatory (CD4+ and CD25/FoxP3+) T-lymphocytes, natural killer cells (NKs), dendritic cells, and macrophages [124]. They were described by Clark et al. [126] in 1969, and their presence in tumoral specimens was subsequently found to be positively associated with better prognosis during the vertical growth phase of high-risk melanoma [127,128,129].

Beyond the dichotomic evaluation of their presence in the tumoral specimen, the following studies have focused on their distribution, phenotype, and state of activation, assuming that these characteristics should reflect the disease’s host reaction and therefore could have an impact on the clinical outcome [130,131,132]. Type, density, and location of immune cells are the variables included in the Immunoscore [133], the predictive role of which was investigated in the MISIPI trial [33]. Surprisingly, CD3+, CD8+, CD20+, CD163+, and FoxP3+ T-cells, both intratumoral (CT) and peritumoral (IM), had no impact on response to treatment in melanoma patients (pts) [33,34,134], underlying the need for functional status analysis of immune cells.

In one of the first biomarker-discovery trials, Hamid et al. [27], enrolled 82 pts with unresectable melanoma (stage IV or III) treated with ipilimumab. Tumor biopsies were performed before the start of treatment and after the second drug administration. Afterwards, pts were divided into a benefit (complete/partial response and stable disease) and non-benefit groups, based on radiological response. In the benefit group, 57.1% of patients had a post-treatment increase in TILs compared to baseline (OR 13.2), and FOXP3+ positivity was detected in 75% of pretreatment samples (vs. 36% in the non-benefit group). Moreover, a high basal indoleamine-2,3-dioxygenase (IDO) expression was related to a favorable outcome. Despite the historically immunosuppressive role of IDO, considerable evidence highlighted that its expression is induced by IFN-γ signaling. From this perspective, a tumoral microenvironment with high IDO expression could reflect an inflamed phenotype [135,136].

In another study, Daud et al. [28] isolated samples of metastatic melanoma before anti-PD-1 therapy and assessed the presence of effector (FOXP3-) and regulatory (FOXP3+) CD4+ T-cells, CD8+ cytotoxic lymphocytes (CTLs), and the expression of PD-1, PD-L1, and CTLA-4 on each cell group. A presence of at least 20% of CTLA-4hi PD-1hi CTLs was related to a greater response rate (RR) (85.7% vs. 0%) and PFS (31.6 months vs. 9.6 months, *p* = 0.017) compared to low CTLA-4hi PD-1hi CTLs. Intriguingly, CTLA-4hi PD-1hi CTLs revealed a partially exhausted phenotype with a maintained production of INF-γ but a reduction of IL-2 and TNF-α. Other T-cell subsets showed no impact on RR.

Consistently, Tumeh et al. [29], in a trial including 46 pts with advanced melanoma treated with pembrolizumab, reported an increase in intratumoral CD8+ cells in pts responsive to the treatment, according to the radiological regression. Regarding the distribution of TILs, CD8+ lymphocytes were more represented at the invasive margin (IM) in pretreatment samples than the non-responder group, suggesting their pivotal role in tumor regression. In the responder group, pembrolizumab also determined an increase of Ki-67-positive CD8+ cells in the tumor center (TC). Conversely, CD4+ cell density, both in TC and IM, showed a low impact on the prediction of treatment response, as previously reported. These data were successively confirmed in the neoadjuvant setting with growing interest in exhausted T-cells and the relation between TILs and circulating lymphocytes [30].

The role of CD4+ lymphocytes might be more important than initially assumed [101]. Indeed, mounting evidence suggests that CD4 lymphocytes could exert a direct and indirect anticancer effect, such as after a vaccination with cancer peptides [101]. Moreover, a trial testing anti-PD-1 drugs in an advanced setting reported a statistically significant association between intratumoral CD4+ cell absolute count and RR [32]. This discordance may be due to the plasticity of CD4+ T lymphocytes, capable of switching from a regulatory to an effector phenotype [132,137]. From this perspective, the ratio between the T-cell populations could provide a more detailed overview of immune balance [132,138]. Of note, CD4 lymphocytes are a heterogeneous class of T-cells [101]. More research is needed to identify the antitumoral effect of each subpopulation.

Natural killer cells (NKs) are usually rare in melanoma tissues, and their predictive and prognostic roles are still debatable [132,139]. Physiologically, NKs are activated by several mediators such as adhesion molecules, soluble extracellular factors, proinflammatory cytokines, and danger-associated molecular patterns (DAMPs) and exert their effector function through the apoptosis of those cells downregulating MHC I molecules, cancer cells included [78,92,132,139].

Preclinical evidence have been emerging, and a high number of NKs seems to predict the response to IT through the production of the FMS-like tyrosine kinase-3 ligand (FLT3L). This hematopoietic cytokine acts as a growth factor [140], which stimulates the intratumoral dendritic cells (DCs) [141]. On the other hand, a small trial found a positive relationship between low circulating total NKs number and ipilimumab response [69]. Intratumoral NKs showed several transcriptional profiles, probably influenced and dynamically regulated by the tumoral microenvironment. Additional studies are required to clarify which subtype may be related to the response to IT and its relationship with NK tumoral levels [142].

Type-2 tumor-associated macrophages (TAMs) are a substantial component of TILs and exert an immune-suppressive function limiting the efficacy of IT through the inhibition of CTLs. Currently, no studies have been conducted in vivo, but preclinical evidence showed promising results with TAM reprogramming strategies [143,144].

### 4.2. Gene Signatures

The host response to the tumor consists of a continuum of innate and adaptive immune reactions, where the immunosurveillance and the immune escape phenomena interplay [145]. Therefore, the quantification and the descriptive analysis of the immune contexture [146] provide a partial overview of the treatment response’s complex mechanism. Those morphological features have been integrated with the underlined genomic landscape analysis, and the role of immune-related genes has been broadly investigated. As a result, the concept of gene-expression profile (GEP) has emerged [147].

INF-γ signature certainly retains the most robust evidence, and several studies have proved its role in establishing an inflamed tumoral microenvironment, both in a palliative and neoadjuvant setting [30,35,148,149].

Ayers et al. [36] conducted a preliminary analysis on 19 pts enrolled in the KEYNOTE-001 trial, building up a 10-gene IFN signature (IFNG, STAT1, CCR5, CXCL9, CXCL10, CXCL11, IDO1, PRF1, GZMA, and MHCII HLA-DRA) which allowed responders to be discriminated from non-responders. This signature was expanded to 28 genes, including some related to antigen presentation, chemokine and cytokine activity, and immunomodulatory factors, and was later validated in a cohort of 62 pts. Both these signatures positively correlated to RR and PFS and confirmed their predictive value to anti-PD-1 therapy. Consistently with the studies reported above, in the OpacinNeo study, a low IFN-γ expression seems to be related to a higher risk of relapse [148].

Besides IFN-γ signature, T-cell-activating genes are involved in response to IT as well.

A pretreatment GEP with high expression of genes related to T-cell infiltration seems to enhance the immune response during the treatment, underlying the importance of a pre-existing inflamed phenotype [150].

In addition to the GEP basal evaluation, the post-treatment genomic modifications have also been evaluated. Both pembrolizumab [30] and ipilimumab [27] were associated with a post-treatment expression of immune-response related genes (such as those coding for immunoglobulins, granzyme B, perforin-1, granulysin, CD8 b-subunit, and T-cell receptor-a and -b subunits) with an impact on recurrence-free survival.

Chen et al. [37] analyzed 46 pts treated with anti-PD-1 agents, progressed to the previous anti-CTLA-4 drug, and investigated their immune profile with an NGS panel. Early on-treatment samples of responders showed 411 significantly differentially expressed genes, predominantly upregulated compared to non-responders, including those coding for chemokines, INF-γ pathway mediators, and adhesion molecules. Moreover, a longitudinal analysis was conducted, and responders to the anti-PD-1 drug revealed an upregulation of 376 genes involved in antigen presentation, T-cell activation, and T-cell homing.

Similarly, Gide et al. [38] performed transcriptomic and immune profiling on 158 melanoma samples and identified two gene clusters associated with better outcomes (PFS) with anti-PD-1 monotherapy. In particular, IFN-related genes (such as TBX21, STAT1, IRF1, TNF, and IFNG) and tumor-infiltrating T-cell genes (CCL5, CXCL13, and IL-2) were highly expressed, suggesting activated T-cells enriched tumoral microenvironment in responders with IFN-secretive phenotype.

Focusing on immune-escape rather than immune-(re)activation, Jiang et al. [151] identified a signature (TIDE) to measure T-cell dysfunction and T-cell exclusion and showed that it could discriminate between responders and non-responders especially in those with high CTL tumoral infiltration.

In conclusion, the emerging evidence on GEP and gene signatures highlights the concept of a genomic (constitutive) predisposition, closely tied to the immune cell infiltration in the tumoral microenvironment. From this perspective, these aspects should be evaluated in combination to provide an exhaustive overview of the complex phenomenon of response to ICIs.

However, predictive gene signatures have not been consistent between different studies, suggesting that there might not exist a unique molecular pattern that predicts response to immunotherapy. From this perspective, an inclusive panel of gene signatures might be able to better predict the outcome from checkpoint inhibitors.

## 5. Systemic Biomarkers

### 5.1. Circulating Factors

Elevated serum lactate dehydrogenase (LDH) is an established surrogate of high tumor burden and an independent adverse prognostic factor in stage IV malignant melanoma patients [152,153]. LDH is included in the eighth edition of the AJCC (American Joint Committee on Cancer) for the malignant melanoma staging system [154]. Several studies proved that patients with an elevated baseline LDH had a significantly shorter overall survival (OS) than patients with normal LDH during ICI therapy [39,40,152]. Moreover, the relative changes in LDH levels during the first ICI treatment weeks are early markers for response and OS [39,40,41]. Preclinical data suggest that LDH, through its interaction in glycolysis and hypoxia mechanisms, contributes to an immune-suppressive microenvironment [155,156]. Even the calcium-binding protein S100B has been used in staging melanoma, establishing prognosis, evaluating treatment success, and predicting relapse [157,158]. High values of S100B and LDH at baseline are associated with the poor outcome either during pembrolizumab alone or with ICI combined therapy [40]. C-reactive protein (CRP), a well-known inflammation index, is another independent prognostic marker in patients with melanoma, associated with shorter OS and melanoma-specific survival [159] and high tumor burden [160]. A recent analysis of CheckMate 064, 066, and 067 trials [42,161] highlighted that higher CRP baseline levels were associated with a weaker response and shorter survival after nivolumab alone, ipilimumab alone, or their combination. A trend towards decreased CRP levels during ipilimumab treatment and response and survival has also been reported [41,160]. Recently, anti-PD-1-treated patients with low serum concentration of hepatocyte growth factor (HGF) showed longer OS and PFS than those with high levels [162]. However, further evidence is needed to determine whether pharmacodynamic changes of these markers can be predictive of treatment efficacy or merely prognostic for survival [40,41,153].

Various cytokines in peripheral blood have been associated with ICI response. Pro-inflammatory IL-6 has been the most studied. High baseline IL-6 levels were associated with a poor response and shorter survival in patients receiving single-agent ICIs [42,48,161]. Moreover, in CheckMate-064, a decrease in IL-6 at 13 weeks was associated with longer OS than those with increasing levels [42]. A drop in IL-8 levels predicted response to anti-PD-1 [49], and baseline serum CXCL5 chemokine levels correlated with objective response to nivolumab in patients with malignant melanoma [50,51]. Furthermore, IL-8 and IL-6 play a role in myeloid-derived suppressor cell (MDSC) recruitment and expansion, further confirming their negative correlation with melanoma patient outcomes [163].

Higher soluble PDL-1 (sPDL-1) levels and sPDL-1 elevation predicted a higher likelihood of clinical benefit after 3 months of pembrolizumab, but they were associated with progressive disease during ipilimumab [52,53]. Recent studies indicated that PD-L1 expression in extracellular vesicles (exosomes), released from both T-cells and dendritic cells isolated from peripheral blood samples, reflects tumor characteristics [164]. Furthermore, PD-L1 is much easier to detect and quantify in circulating exosomes rather than in biopsies or when soluble in plasma [55]. Baseline increased PD-L1 and CD28 exosomal protein expression was associated with improved clinical response, PFS, and OS in ipilimumab-treated MM patients [54]. Moreover, a high increase in exosomal PD-L1 expression could predict tumor progression to ICI therapy [55].

Finally, soluble CTLA-4 levels were reported to be a positive predictive factor for ipilimumab therapy and to correlate with the best overall response [56].

Although promising, these blood-based biomarkers require large-scale prospective validation and cutoff definition.

### 5.2. Circulating Lymphocytes

Some insights in predicting survival and response during ICI therapy come from peripheral T-cell phenotype assessment. Higher absolute or relative lymphocyte, eosinophil, and neutrophil count at baseline and their increase during ICI treatment were associated with disease control and survival [41,57,58,59,60,165]. Higher baseline peripheral blood neutrophil to lymphocyte ratio (NLR), which may reflect chronic systemic inflammation, was strongly and independently associated with decreased PFS and OS with ICIs in several studies [42,61,62,63,64,65,66,67]. Alongside the simple absolute value and lymphocyte ratios, various T-cell phenotypes were investigated.

High frequencies of baseline CD8 effector-memory type 1 T-cells and normal baseline CD45RO+CD8+ T-cells levels correlate with longer OS (and only the first also higher clinical response rates) during ipilimumab [68,69].

Regulatory T-cells (Tregs), formerly known as suppressor T-cells, represent direct target cells of ipilimumab due to their constitutive CTLA-4 expression. Accordingly, baseline high FoxP3-Treg frequencies and their drop during ipilimumab were significantly associated with better survival [41,59]. An early increase in peripheral-blood Tregs and decreased antigen-specific T-cells have been associated with progression during nivolumab plus vaccine therapy [70].

Moreover, CD37 expression on circulating CD8+PD-1+ lymphocytes, involved in immune-suppressive adenosine production, seems to be a promising marker of clinical response to nivolumab [166]. Likewise, a lower than the median frequency of peripheral blood PD-1+CD56+ T-cells before starting anti-PD-1 is related to superior clinical response, longer PFS, and OS of stage IV melanoma patients [71].

Finally, an increase in relative numbers of CD4 and CD8 memory T-cell subsets and functionally active natural killer subsets were described as potential biomarkers for response to anti-CTLA-4 and anti-PD-1, respectively [72,165].

As it is known, prolonged and repeated antigen stimulation causes the T-cell exhaustion, consisting of loss of effector functions (such as IFNgamma production) and expression of multiple surface inhibitory receptors such as PD-1 and CTLA-4 [167,168]. Although it has been proposed as a promising biomarker when studied in TILs [168], peripheral blood expression of T-cell exhaustion markers does not distinguish between responders and non-responders to anti-PD-1 [73].

Finally, it is known that through the MHC-antigen complex-TCR interaction, a naïve T-cell is activated and clonally expanded. Therefore, the repertoire of T-cell receptors (TCRs) reflects the sum of previous exposures of unique antigens to the host. Intratumoral expanded TCR clonality at baseline and on-treatment was associated with response to ICIs [15,29]. Similarly, CTLA-4 and PD-1 blockade increased peripheral blood mononuclear cell TCR diversity as reflected in the higher number of unique TCR clonotypes in responders vs. non-responders [70,74,169]. However, conflicting results have been reported [11], and TCR analysis data seem more robust in tumoral specimens.

### 5.3. Microbiota

The human gastrointestinal tract harbors a number exceeding 1014 of symbiotic microorganisms, the gut microbiota, which exerts a marked influence on the host during homeostasis and disease [170]. There is growing evidence that gut microbiota composition and, in particular, specific bacterial species influence the efficacy and toxicity of ICI therapy in patients with metastatic melanoma [22,23,25]. An increased microbiota diversity, irrespective of species identity, was associated with improved ICI response [25,171]. Notably, baseline *Faecalibacterium* spp. enriched gut microbiota correlates with high peripheral blood CD4+/CD8+ effector T-cells, CD8+ TILs, and with a maintained cytokine response, and this translates into a better clinical response (PFS and OS) to ipilimumab, anti-PD-1, and ICI combination therapy in metastatic melanoma patients [23,24,25,172]. Albeit with some contrasting evidence, *Bacteroides* spp. seem to be associated with a reduced inflammatory response (high Treg levels, limited inflammatory cytokine concentrations) to ICIs [23,173].

Microbiota plays a role in immunomodulation through bacterial metabolites. Short-chain fatty acid (SCFA) serum levels negatively correlate with anti-CTLA-4 efficacy in metastatic melanoma patients [24]. Conversely, elevated fecal SCFAs have recently been related to better outcomes in a patient with metastatic melanoma and other solid tumors treated with anti-PD-1 therapy [26]. Notably, the composition of gut microbiota also seems to be associated with ICIs-induced colitis [23,174].

### 5.4. Others

As no diagnostic companions have been approved for ICI therapy in melanoma, relevant efforts have been made by the scientific and medical community to find possible biomarkers for therapy response. Among candidate biomarkers, gene-expression profiles and methylation patterns have shown possible utility in predicting therapeutic response. In pretreatment melanoma samples from patients receiving anti-PD-1 therapy, PD-L2 promoter DNA hypomethylation and high PD-L2 mRNA expression were shown to predict more prolonged progression-free survival and overall survival [175]. Gupta and colleagues reported a four-gene multiplex mRNA panel targeting PD-L1, PD-L2, CD8A, IRF1, and combined PD-L1/PD-L2 mRNA levels with promising associations with checkpoint inhibition outcomes [176]. From a recent in silico analysis of mRNA expression profiling, IRF1 and CD8A together with JAK2 and SELL have been shown to predict response to anti-PD-1 immunotherapy in melanoma patients [177]. Regarding anti-CTL4A drugs, granzyme A (GZMA) and perforin (PRF1) mRNA expression levels in pretreatment melanoma biopsies were significantly enriched in patients experiencing a clinical benefit from ipilimumab. Both transcripts have been shown to correlate with the local immune infiltrate and neoantigen load [7].

Besides TMB, mutations at specific genes seemed to predict response to ICI therapy in melanoma. Patients whose melanomas are NRAS mutated seem to respond to immunotherapy better and may have better outcomes than other genetic subtypes, suggesting that ICI therapy may be particularly effective as a treatment option for NRAS-mutant melanoma [178]. Among less frequent mutations, in two independent cohorts of patients, mutations in Serpin B3 or Serpin B4 genes have been shown to define a subgroup of patients who responded to anti-CTL4A therapy and had longer overall survival [179]. In contrast, mutations to low-density lipoprotein receptor-related protein 1B (LRP1B), as a possible surrogate biomarker for TMB, were significantly associated with a better checkpoint inhibition survival outcome [180]. Noncoding RNAs have also been investigated as possible predictive biomarkers. In a small sample cohort, higher miR-222 levels were associated with a lack of benefit to anti-CTL4 therapy [181]. In another recent study, a score of long non-coding RNAs was associated with immunotherapeutic overall survival benefit both in the IMvigor210 trial cohort (AUC, 0.79 at 12 months and 0.77 at 20 months) and in the TCGA melanoma cohort [182].

### 5.5. MDSCs

Myeloid-derived suppressor cells are a heterogeneous class of immature myeloid-suppressive cells [183]. Firstly, a reported strong and independently validated predictor of PFS and OS in response to anti-PD-1 was pretreatment monocytes levels rather than their gene-expression patterns or polarization (e.g., MDSCs differentiation) [57,76]. Afterward, low frequencies of MDSCs and their decline compared to baseline values were related to good prognosis and clinical benefit to ICI therapy [57,77,78,184].

### 5.6. ctDNA

Liquid biopsy and circulating tumor DNA (ctDNA) are increasingly integrated into clinical practice. Detectable ctDNA turned out to be associated with higher tumor burden and visceral metastases [82]. The absence of measurable ctDNA at baseline or first evaluation was an independent predictor of response and survival in PD-1 antibody-treated patients [79]. Moreover, high TMB evaluated by liquid biopsy and a >50% decrease of cell-free DNA concentration or undetectable ctDNA at three weeks after combined CTLA-4 and PD-1 antibody therapy initiation seemed to be associated with better response and OS [80].

In a recently published study exploring ctDNA evaluation, pretreatment ctDNA is a reliable and validated indicator of longer PFS for patients with metastatic melanoma in the first-line ICI treatment setting, but not in the second-line context, especially in those pretreated with BRAF/MEK inhibitors [81]. In a small cohort of metastatic melanoma patients, ctDNA assessments indicated evidence of melanoma activity that early predicted radiographic evidence of disease progression [82]. An appealing application is the longitudinal ctDNA evaluation in the challenging differentiation of pseudoprogression from real disease progression during ICI therapy. In a cohort study of 125 patients with metastatic melanoma treated with anti-PD-1 alone or in combination with ipilimumab, a reduction of at least 10-fold within the first evaluation of the number of ctDNA copies accurately identified patients with pseudoprogression. A favorable ctDNA profile, defined as undetectable baseline ctDNA that remained undetectable or detectable ctDNA at baseline that became undetectable or decreased by at least 10-fold during treatment, was significantly associated with improved OS [83]. An intriguing application is under investigation in the ongoing CAcTUs clinical trial [NCT03808441]; the longitudinal ctDNA evaluation is used to guide the switch between targeted therapy and immunotherapy in advanced cutaneous melanoma patients.

Taken together, this evidence suggests that ctDNA assessment, while not useful for “a priori” patient selection, could be an extremely promising non-invasive real-time tumor burden monitoring tool for early assessment of response to ICI therapy.

## 6. Conclusions

Immunotherapy has revolutionized the therapeutic landscape of melanoma. In particular, checkpoint inhibition has been shown to increase long-term outcomes, and, in some cases, it can be virtually curative. However, the absence of clinically validated predictive biomarkers is one of the major causes of the unpredictable efficacy of immunotherapy. Nevertheless, several putative predictive biomarkers have been proposed, but it seems that a single factor could not summarize the entire complexity of cancer. Indeed, none of the abovementioned biomarkers possess high sensitivity and specificity when used alone. While TMB, TILs, and PD-1 could have predictive power, it appears that a combination of multiple biomarkers could better predict the efficacy of immunotherapy. This hypothesis has culminated in the concept of a “cancer immunogram”, which accounts for several parameters that combined could estimate the efficacy of immunologic treatments [185]. In line with this concept, some interesting composite predictive tools have been developed [13,151,186,187,188]. However, in order to demonstrate an effective predictive value and a clinical utility, these composite scores should be tested prospectively. In addition, immune-related side effects might serve as an early surrogate of immunotherapy efficacy. For example, immune-induced vitiligo was shown to be linked to a favorable prognosis [189].

In perspective, liquid biopsy, ctDNA, and other circulating biomarkers need to complete development to be integrated in clinical practice with the aim to predict immunotherapy response. In addition, biomarkers might show utility also for differentiating cancer progression versus pseudoprogression.

In conclusion, it could be anticipated that in the next few years, an increasing number of useful composite predictive biomarkers tools will be available, guiding clinicians to a better stratification of patients, choice of alternative treatments to immune checkpoints, and rational de-escalation/escalation of cancer treatments. However, a deep knowledge of single biomarkers is of pivotal importance to understand why and how they should be combined.

## Figures and Tables

**Table 1:** List of predictive biomarkers for immunotherapy in melanoma.

Biomarker	Study Details & Reference	Patients	Clinical Utility
TMB (WES)	Ipilimumab Van Allen et al. [7]	110	CB *
TMB (WES)	Ipilimumab or tremelimumab Snyder et al. [8]	64	OS
TMB (WES)	Nivolumab or pembrolizumab Hugo et al. [9]	38	OS
TMB (NGS)	Nivolumab or pembrolizumab or atezolizumab Johnson et al. [10]	65	RR, PFS, OS
TMB (WES)	Nivolumab and ipilimumab Weber et al. [11]	94	RR for the sequence: Nivo → Ipi
TMB (WES)	Nivolumab Riaz et al. [12]	68	OS
TMB (WES)	Pembrolizumab Cristescu et al. [13]	89	RR
TMB (NGS)	Ipilimumab or adoptive T-cell therapy Rosizik et al. [14]	76	PFS, OS
TMB (WES)	Anti-PD-1 or anti-CTLA-4 Roh et al. [15]	21	RR (numerical)
TMB (NGS)	Anti-PD-1 and/or anti-CTLA-4 Morrison et al. [16]	160	RR
MHC-II	Nivolumab or pembrolizumab Liu et al. [17]	32	RR (in previously exposed to anti-CTLA-4)
MHC-II	Anti-PD-1 Johnson et al. [18]	30	RR, PFS, OS
MHC-II	Nivolumab or pembrolizumab Johnson et al. [19]	166	RR, PFS
MHC-I MHC-II	Ipilimumab and/or nivolumab Rodig et al. [20]	181	OS (MHC-I for ipilimumab; MHC-II for nivolumab)
B2M	Anti-CTLA-4 or anti-PD-1 Sade-Feldman et al. [21]	143	RR, OS
Gut Microbiota	Anti-CTLA-4 Vetizou et al. [22]	25	Antitumor response
Gut Microbiota	Anti-CTLA-4 Chaput et al. [23]	26	PFS, OS
Gut Microbiota	Anti-CTLA-4 Coutzac et al. [24]	85	PFS, OS
Gut microbiota	anti-PD-1 Gopalakrishnan et al. [25]	112	ORR, PFS
Gut microbiota	Anti-PD-1 Nomura et al. [26]	52	ORR, PFS
TILs	Anti-CTLA-4 Hamid et al. [27]	82	RR
TILs	Anti-PD-1 Daud et al. [28]	20	RR, PFS
TILs	Anti-PD-1 Tumeh et al. [29]	46	RR
TILs	Anti-PD-1 Huang et al. [30]	27	PCR, DFS
TILs	Anti-PD-1 or Anti-PD-1 + anti-CTLA-4 Amaria et al. [31]	23	ORR
TILs	Anti-PD-1 Uryvaev et al. [32]	30	RR, PFS
Immunoscore	Anti-CTLA-4 Bifulco et al., Galon et al. [33,34]	190	No relationship with RR
IFN signature	Anti-PD-1 Karachaliou et al. [35]	21	DFS, PFS, OS
IFN signature	Anti-PD-1 Ayers et al. [36]	19	RR, PFS
Expanded immune signature	Anti-PD-1 Ayers et al. [36]	62	RR, PFS
Immune signature	Anti-PD-1 Chen et al. [37]	46	RR
Immune signature	Anti-PD-1 Huang et al. [30]	27	RFS
Immune signature	Anti-PD-1 Gide et al. [38]	158 (samples)	PFS
LDH	Anti-PD-1 Diem et al. [39]	66	OS, ORR
LDH	Anti PD-1 or anti-CTLA-4 Wagner et al. [40]	238	OS
LDH	Anti-CTLA-4 Simeone et al. [41]	95	OS, RR
S100B	Anti PD-1 or anti-CTLA-4 Wagner et al. [40]	238	OS
PCR	Anti-PD-1 end/or anti-CTLA-4 Laino et al. [42] Wagner et al. [40]	1503	OS, RR
PCR	Anti-CTLA-4 Simeone et al. [43]	95	OS, RR
PCR	Anti-CTLA-4 Nyakas et al. [44]	69	OS
HGF	Anti-PD-1 Kubo et al. [45]	29	OS, PFS
IL-6	Anti-PD-1 end/or anti-CTLA-4 Laino et al. [46] Wagner et al. [47]	1503	OS, RR
IL-6	Anti-CTLA-4 Valpione et al. [48]	140	OS
IL-8	Anti-PD-1 ± anti-CTL-4 Sanmamed et al. [49]	29 + 34 validation cohort NSCLC + melanoma pts)	OS, RR
CXCL-5	Anti-PD-1 Fujimura et al. [50,51]	46	ORR
sPDL-1	Anti-PD-1 Dronca et al. [52]	60	ORR
sPDL-1	Anti-CTLA-4 or anti-PDL-1 Zhou et al. [53]	90	ORR
PD-L1 and CD28 exosomal expression	Anti-CTLA-4 Tucci et al. [54]	59	CR, PFS, OS
Exosomal PD-L1	Anti-PDL-1 and/or anti-CTLA-4 Cordonnier et al. [55]	100	RR, PFS, OS
CTLA-4	Anti-CTLA-4 Pistillo et al. [56]	113	ORR
Absolute lymphocyte count	Anti-CTLA-4 Simeone et al. [41]	95	OS, RR
Relative eosinophil and lymphocyte count	Anti-PDL-1 Weide et al. [57]	616	OS
Eosinophil and lymphocyte count	Anti-CTLA-4 Delyon et al. [58]	73	OS
Absolute lymphocyte count	Anti-CTLA-4 Martens et al. [59]	82	OS
Absolute monocyte/eosinophil and relative lymphocyte counts	Anti-CTLA-4 Martens et al. [59]	209	OS, RR
Absolute lymphocyte and neutrophil count	Anti-PDL-1 Nakamura et al. [60]	98	OS
NLR	Anti-PD-1 end/or anti-CTLA-4 Laino et al. [42]	1503	OS, RR
NLR	Anti-CTLA-4 Ferrucci et al. [61]	69	PFS, OS
NLR	Anti-CTLA-4 Ferrucci et al. [62]	720	PFS, OS
NLR	Anti-PD-1 Capone et al. [63]	97	PFS, OS
NLR	Anti-PD-1 Bartlett et al. [64]	224	TTF, OS
NLR	Anti-CTLA-4 Zaragoza et al. [65]	58	OS
NLR	Anti-CTLA-4 Cassidy et al. [66]	197	RR, PFS, OS
NLR	Anti-PD-1 Fujisawa et al. [67]	90	ORR
CD8 effector-memory type 1 T-cells	Anti-CTLA-4 Wistuba-Hamprecht et al. [68]	137	OS, RR
CD45RO+CD8+ T-cells	Anti-CTLA-4 or anti-PD-1 Tietze et al. [69]	30	OS
FoxP3-Tregs	Anti-CTLA-4 Simeone et al. [41]	95	OS, RR
FoxP3-Tregs	Anti-CTLA-4 Martens et al. [59]	209	OS, RR
FoxP3-Tregs	Nivolumab with vaccine Weber et al. [70]	90	ORR
CD37	Anti-PD-1 Capone et al. [63]	100	ORR, OS
PD-1+CD56+ T-cells	Anti-PD-1 Bochem et al. [71]	75	PFS, OS, RR
CD4 and CD8 memory T- cells	Anti-CTLA-4 Martens et al. [59]	82	OS
NK T-cells	Anti-PD-1 Subrahmanyam et al. [72]	67	ORR
T-cell exhaustion markers	Anti-PD-1 Pirozyan et al. [73]	42	No differences in RR
TCR repertoire	Nivolumab with vaccine Weber et al. [70]	90	ORR
TCR repertoire	Anti-CTLA-4 Cha et al. [74]	21 melanoma pts	ORR
TCR repertoire	Anti-CTLA-4 Postow et al. [75]	12	CB *
Monocyte frequency	Anti-PD-1 Weide et al. [57]	616	OS
Monocyte frequency	Anti-PD-1 Krieg et al. [76]	60 samples	PFS, OS
MDSCSs	Anti-PD-1 Weide et al. [57]	616	OS
MDSCSs	Anti-PD-1 De Coana et al. [77]	43	ORR, OS
MDSCSs	Anti-CTLA-4 Meyer et al. [78]	49 (+15 controls)	ORR
MDSCSs	Anti-CTLA-4 Weber et al. [11]	92	ORR, PFS, OS
ctDNA	Anti-PD-1 (± anti-CTLA-4) Lee et al. [79]	86	RR, PFS, OS
TMB, ctDNA, cell-free DNA	Anti-PD-1 or anti-CTLA-4 Forschner et al. [80]	35	ORR, OS
ctDNA	Anti-PD-1 Marsavela et al. [81]	125 patients discovery cohort + 128 validation samples	PFS, OS
ctDNA	Anti-PD-L1 and/or anti-CTLA-4 or BRAF/MEK inhibitors Rowe et al. [82]	127	ctDNA mutant fraction and sum of tumor diameters relationship
ctDNA	Anti-PD-1 (± anti-CTLA-4) Lee et al. [83]	125	ORR, OS

CB: clinical benefit; ctDNA: circulating tumor DNA; Ipi: ipilimumab; MDSCSs: myeloid derived suppressor cells; NGS: next-generation sequencing; Nivo: nivolumab; NLR: neutrophil to lymphocyte ratio; OS: overall survival; TMB: tumor mutational burden. * defined as evidence of tumor burden reduction or prolonged stable disease lasting at least 9 months.

**Table 2:** ORR in PD-L1-positive and -negative strafied by treatment type.

References	Treatment	Mean ORR in PD-L1-Positive (SD, 95% CI)	Mean ORR in PD-L1-Negative (SD, 95% CI)	*p* * Value
[43,46,47,110,111,118,119,121,125]	Pembrolizumab	41.3 (23.2, 24.8–57.9)	23.0 (17.7, 10.4–35.7)	0.009
[75,112,118,125]	Ipilimumab	22.6 (11.5, 4.4–40.9)	10.0 (4.3, 3.2–16.8)	0.1
[43,46,70,110,113,116,117,120]	Nivolumab	47.5 (9.6, 40.1–54.9)	24.0 (19.5, 8.9–39.0)	0.01
[43,118]	Nivolumab + ipilimumab	65.2 (9.7, -22.0–152.4)	55.5 (0.3, 53.3–57.8)	0.4
§ [16,19,44,45,110,114,115]	Combined therapy and atezolizumab (1 study)	46.2 (19.8, 29.6–62.7)	27.7 (18.6, 12.1–43.2)	0.0008
Overall results based on a total of 5069 patients		44.1 (19.0, 37.3–50.9)	24.5 (18.5, 18.0–31.0)	<0.0001

§ Treatments include combinations of nivolumab and pembrolizumab (2 studies); ipilimumab and pembrolizumab (1 study); treatment with nivolumab or ipilimumab or their combination (1 study); nivolumab or pembrolizumab (2 studies); atezolizumab (1 study), nivolumab and other treatments (1 study). * *t*-test for paired data. ORR: objective response rate; SD: standard deviation; CI: confidence interval.

## Supplementary Materials

The following are available online at https://www.mdpi.com/article/10.3390/cancers13081819/s1, Supplementary data 1.

## Abbreviations

CI	confidence interval
ctDNA	circulating tumor DNA
ICIs	immune-checkpoint inhibitors
IHC	Immunohistochemistry
LDH	lactate dehydrogenase
MDSC	myeloid derived suppressor cells
MHC	major histocompatibility complex
MM	metastatic melanoma
NGS	next-generation sequencing
NLR	neutrophil to lymphocyte ratio
ORR	objective response rate
RR	response rate
SD	standard deviation
TCR	T-cell receptor
TMB	tumor mutational burden
