# Supplementary material for: Find the Flame: Predictive Biomarkers for Immunotherapy in Melanoma
Source: Cancers (Basel). 2021 Apr 10;13(8):1819. doi: 10.3390/cancers13081819 (PMC8070445; doi:10.3390/cancers13081819)
Supplement: Supplementary file 1 [file cancers-13-01819-s001.pdf]

Searching details:

We interrogated the database PubMed using the following keywords: (1) PD-L1 expression melanoma immunotherapy response; (2) melanoma keynote; (3) PD-L1 immunohistochemistry melanoma predictive; (4) PD-L1 nivolumab response melanoma; (5) PD-L1 expression predictive immunotherapy melanoma. We searched articles until November 2020. The reference lists were also carefully checked to identify additional eligible studies. All analyses were carried out with previously published data, consequently no ethical approval or patient consent were included in this study.

Selection Criteria

Studies were included if they met the following criteria: (1) inclusion of patients diagnosed with confirmed melanoma; (2) treatment with immune checkpoint inhibitors; (3) detection of PD-L1 expression in the melanoma tissue, including also metastases, by immunohistochemistry (IHC) irrespective of the antibody clone used; (4) identification of a definite cut-off value in the analysis of PD-L1 expression; (5) availability of RECIST data (responders = complete + partial response according to RECIST for solid tumors) in PD-L1 positive and in PD-L1 negative patients; (5) publications in English language. Determination of PD-L1 expression in melanoma was based in most analyzed studies by the percentage of PD-L1 expressing tumor cells, however in a minority of studies also inflammatory cells in nest of tumor cells were counted.

The exclusion criteria were as follows: (1) case reports, reviews (systematic reviews were retained), letters, and correspondences; (2) studies without available or usable information; (3) studies including adjuvant therapy with immune checkpoint inhibitors; (4) studies lacking PD-L1 or RECIST data; (5) animal studies; (6) publications in languages different from English; (7) duplicate studies.

Data Extraction

One researcher (SB) extracted basic information from the included studies, analyzing also supplementary data where available. The following data were extracted from eligible studies: the first author's name, publication year, sample size, detection method, antibody used (where available), treatment, cut-off values, number of responders and non-responders in the group of PD-L1 positive and PD-L1 negative patients.

Statistical Analysis

The percent of responders and non-responders in the group of PD-L1 positive and PD-L1 negative were used to set-up the meta-analysis for binary data. The association between PD-L1 expression and response to ICI therapy was assessed by odds ratios (ORs) and their 95% CIs. I^2 metric was used to inspect the statistical heterogeneity of the data. A P value of less than 0.1 or an I^2 value of more than 50% indicated significant heterogeneity, and a random effects model was employed for calculation. All statistical analyses were carried out using Stata version 16.0 (STATA Corp., College Station, TX). $P < 0.05$ was considered to indicate statistical significance.

Results

In PubMed, after removal of duplicates, were retained 309 publications. From these, 52 articles were read as full-text. From those ones were retained 22 articles as they fulfilled the inclusion criteria.
